# Cholesteatoma Fibroblasts Promote Epithelial Cell Proliferation through Overexpression of Epiregulin

**DOI:** 10.1371/journal.pone.0066725

**Published:** 2013-06-24

**Authors:** Mamoru Yoshikawa, Hiromi Kojima, Yuichiro Yaguchi, Naoko Okada, Hirohisa Saito, Hiroshi Moriyama

**Affiliations:** 1 Department of Otorhinolaryngology, Toho University School of Medicine, Meguro-ku, Tokyo, Japan; 2 Department of Otorhinolaryngology, Jikei University School of Medicine, Minato, Tokyo, Japan; 3 Department of Allergy and Immunology, National Research Institute for Child Health and Development, Okura, Setagaya-ku, Tokyo, Japan; University of Tennessee, United States of America

## Abstract

To investigate whether keratinocytes proliferate in response to epiregulin produced by subepithelial fibroblasts derived from middle ear cholesteatoma. Tissue samples were obtained from patients undergoing tympanoplasty. The quantitative polymerase chain reaction and immunohistochemistry were performed to examine epiregulin expression and localization in cholesteatoma tissues and retroauricular skin tissues. Fibroblasts were cultured from cholesteatoma tissues and from normal retroauricular skin. These fibroblasts were used as feeder cells for culture with a human keratinocyte cell line (PHK16-0b). To investigate the role of epiregulin in colony formation by PHK16-0b cells, epiregulin mRNA expression was knocked down in fibroblasts by using short interfering RNA and epiregulin protein was blocked with a neutralizing antibody. *Epiregulin* mRNA expression was significantly elevated in cholesteatoma tissues compared with that in normal retroauricular skin. Staining for epiregulin was more intense in the epithelial cells and subepithelial fibroblasts of cholesteatoma tissues than in retroauricular skin. When PHK16-0b cells were cultured with cholesteatoma fibroblasts, their colony-forming efficiency was 50% higher than when these cells were cultured with normal skin fibroblasts. Also, knockdown of epiregulin mRNA in cholesteatoma fibroblasts led to greater suppression of colony formation than knockdown in skin fibroblasts. Furthermore, the colony-forming efficiency of PHK16-0b cells was significantly reduced after treatment with an epiregulin neutralizing antibody in co-culture with cholesteatoma fibroblasts, but not in co-culture with skin fibroblasts. These results suggest that keratinocyte hyperproliferation in cholesteatoma is promoted through overexpression of epiregulin by subepithelial fibroblasts via epithelial–mesenchymal interactions, which may play a crucial role in the pathogenesis of middle ear cholesteatoma.

## Introduction

Middle ear cholesteatoma is caused by the hyperproliferation of keratinocytes due to chronic inflammation and it is one of the hyperproliferative epithelial diseases such as psoriasis and acanthoma. Cholesteatoma is a benign keratinizing hyperproliferative epithelial lesion of the petrous temporal bone that can invade the surrounding bone and middle/inner ear structures. In patients with advanced cholesteatoma, erosion of the ossicles and otic capsule can result in hearing loss, vestibular dysfunction, facial paralysis, and even intracranial complications [1]. The only effective treatment is complete surgical eradication of the cholesteatoma, but postoperative recurrence is unfortunately very common.

Although the molecular mechanisms involved in the pathogenesis of cholesteatoma are not yet fully understood, it has been suggested that keratinocyte proliferation and migration are mediated by several autocrine and paracrine growth factors and their receptors [2], [3]. Recently, it was proposed that interactions between mesenchymal cells such as fibroblasts and epithelial cells or keratinocytes are involved in the processes of inflammation, homeostasis, and tissue regeneration [4], [5], [6], [7], [8]. For example, interleukin (IL)-1 produced by keratinocytes induces fibroblasts to secrete keratinocyte growth factor (KGF), granulocyte macrophage-colony stimulating factor (GM-CSF), and transforming growth factor (TGF)-α [9], [10], [11], and these fibroblast-derived cytokines support the proliferation and differentiation of keratinocytes. This paracrine loop is thought to be important in the process of tissue repair after inflammation.

We previously identified many differences in the pattern of gene expression between retroauricular skin fibroblasts (SF) and middle ear cholesteatoma fibroblasts (MECF) [12]. For example, the expression of *LARC*, *GMCSF*, *epiregulin*, *ICAM1*, and *TGFA* mRNA was significantly more up-regulated in MECF than in SF by IL-1α and/or IL-1β stimulation with spontaneous expression of *epiregulin* and *TGFA* being substantially enhanced in particular. Such differential gene expression suggests that subepidermal fibroblasts may play a role in the occurrence of hyperkeratosis during the growth of cholesteatoma by releasing molecules involved in inflammation and epidermal cell proliferation.

Epiregulin is a member of the EGF family that includes EGF, TGF-α, heparin-binding EGF, amphiregulin, betacellulin, neuregulin, and tomoregulin. Among these molecules, epiregulin, heparin-binding EGF, and betacellulin are known to bind to ErbB4 as well as to the epidermal growth factor receptor (EGFR)/ErbB1 [13], [14], [15]. Together with these receptors, epiregulin regulates cell differentiation, growth, and homeostasis, so their role in cancer and skin disease has been intensively examined [16], [17], [18]. It has been reported that epiregulin is not detected in normal human fibroblasts, but is expressed by human fibroblasts that have been immortalized by telomerase reverse transcriptase [19] or by fibroblastic cells in malignant fibrous histiocytoma [20].

Among the various growth factors that could play a role in the development of cholesteatoma, KGF was considered likely to be a key effector [3], [21], [22]. However, we previously found no significant difference of KGF gene expression between SF and MECF by DNA microarray analysis [12]. This suggested that fibroblasts might play a role in the development of hyperkeratosis during the pathogenesis of middle ear cholesteatoma through overexpression of epiregulin rather than KGF.

However, there have been no reports about the role of epiregulin in cholesteatoma. Accordingly, we performed the present study to investigate whether keratinocytes underwent proliferation when exposed to epiregulin produced by MECF or SF.

## Materials and Methods

### Sample Preparation

Middle ear cholesteatoma tissues and normal retroauricular skin samples were obtained from 10 patients undergoing tympanoplasty at Jikei University Hospital (Tokyo, Japan). The study was approved by the Jikei university ethics review board prior to commencement of the study, and written informed consent was obtained from all subjects.

RNAlater (Qiagen, Hilden, Germany) was used to preserve one part of each specimen of cholesteatoma or retroauricular skin tissue for examination of epiregulin mRNA expression. The other part of each specimen was fixed overnight in 10% buffered formalin at room temperature and embedded in paraffin to provide sections for immunohistochemical analysis of epiregulin, epidermal growth factor receptor (EGFR)/ErbB1, and ErbB4 expression.

### Cell Culture

Residual tissue pieces were minced into single cells, washed with phosphate-buffered saline (PBS), and then incubated in DMEM/F12 (Life Technologies, Carlsbad, CA) with 10% fetal calf serum (FCS) (JRH Bioscience, Lenexa, KS) and 100 U/ml penicillin-100 µg/ml streptomycin (Life Technologies) for several weeks. As a result, fibroblasts derived from cholesteatomas (MECF) and fibroblasts derived from normal retroauricular skin (SF) were obtained. These cells were incubated at 37°C in a humidified incubator under 5% CO_2_ in air and were analyzed after 4 passages.

A human keratinocyte cell line (PHK16-0b) was obtained from the Health Science Research Resources Bank (HSRRB; Osaka, Japan), and was maintained in defined keratinocyte serum-free medium (KSFM; Life Technologies) with 10 ng/mL recombinant human epithelial growth factor, 1% penicillin/streptomycin, and the growth supplement supplied by the manufacturer at 37°C under 5% CO_2_. Human epidermal keratinocytes (HEK) derived from normal adult skin were purchased from Kurabo (Osaka, Japan) and were cultured in keratinocyte basal medium (HuMedia-KG2; Kurabo).

### Quantitative Real-time Polymerase Chain Reaction (PCR)

Total RNA was isolated by using an RNeasy Mini Kit that included DNase (Qiagen) and the RNA was transcribed to obtain cDNA using superscript II (Life Technologies). Then quantitative PCR was performed with an ABI/PRISM 7700 Sequence Detector System (Applied Biosystems, Foster City, CA) and SYBR green PCR master mix (Life Technologies). The primers for epiregulin were as follows: forward 5′- ATCCTGGCATGTGCTAGGGT-3′, reverse 5′- GTGCTCCAGAGGTCAGCCAT-3′. The level of target mRNA expression was normalized by the average expression of the housekeeping gene GAPDH.

### Immunohistochemistry

Immunohistochemistry was performed with a monoclonal antibody for epiregulin (183625; R&D systems, Minneapolis, MN), a monoclonal antibody for EGFR (102618; R&D systems), and a polyclonal antibody for ErbB4 (C-18; Santa Cruz Biotechnology, Santa Cruz, CA). Serial sections were cut at a thickness of 4 µm and were placed onto glass slides, followed by deparaffinization for 10 minutes at 37°C. An Envision™ kit (Dako, Glostrup, Denmark) was used for detection. The blocking solution was applied for 10 minutes, followed by incubation for 1 hour with the primary antibodies. After incubation with the secondary antibody for 30 minutes, DAB was applied for 5 minutes to detect the reaction products for epiregulin, EGFR, and ErbB4. Finally, the sections were counterstained, dehydrated, and coverslipped.

### Cell Proliferation Assay

PHK16-0b cells were seeded into a 96-well flat-bottomed tissue culture plate. After 72 h of incubation with recombinant epiregulin (0, 1, 10, or 100 ng/mL) with or without 5 µM of the EGF receptor tyrosine kinase inhibitor AG-1478 (Calbiochem, San Diego, CA), cell viability was evaluated with a Cell Count Reagent SF colorimetric assay (Nacalai Tesque, Kyoto, Japan). Briefly, 10 µl of Cell Count Reagent SF was added to each well and incubation was done for 2 h at 37°C. Then viable cells were assessed by measuring the OD at 450 nm using a microplate reader (Wallac ARVO SX 1420 Multilabel Counter; Perkin Elmer, Norwalk, CT). Cells cultured without epiregulin treatment served as the control.

### Colony-Forming Assay

MECF or SF were incubated in DMEM/F12 containing 10% FCS and were plated into 12-well culture plates. On the next day, the cells were treated with mitomycin C (Nacalai Tesque) for 3 hours, and single PHK16-0b or HEK cells were then seeded at a density of 300/well in defined KSFM. The colony-forming efficiency (CFE) was evaluated on day 21 after the cells were fixed with formalin and stained with 1% rhodamine B (Wako, Osaka, Japan).

Synthetic Stealth siRNA for human epiregulin (Life Technologies) was used to knockdown epiregulin mRNA expression in the feeder cells. MECF or SF at 50% confluence were transfected for 24 hours with the siRNA oligonucleotide using Lipofectamine RNAiMAX (Life Technologies) according to the manufacturer's instructions. Stealth siRNA Negative Control Duplexes (Life Technologies) was used as the negative control.

To block epiregulin protein expression on the cell surface and in culture supernatant during co-culture, an epiregulin neutralizing antibody (Santacruz) or a control antibody was added to the wells for the first 7 days.

### Statistical Analysis

Data are presented as the mean ± standard error of the mean (SEM). Statistical significance was determined by using the paired Student’s *t-*test, and differences were considered significant at *p*<0.05.

## Results

### Elevated Expression of Epiregulin Mrna and Protein in Middle Ear Cholesteatoma Tissue

**Figure 1:**
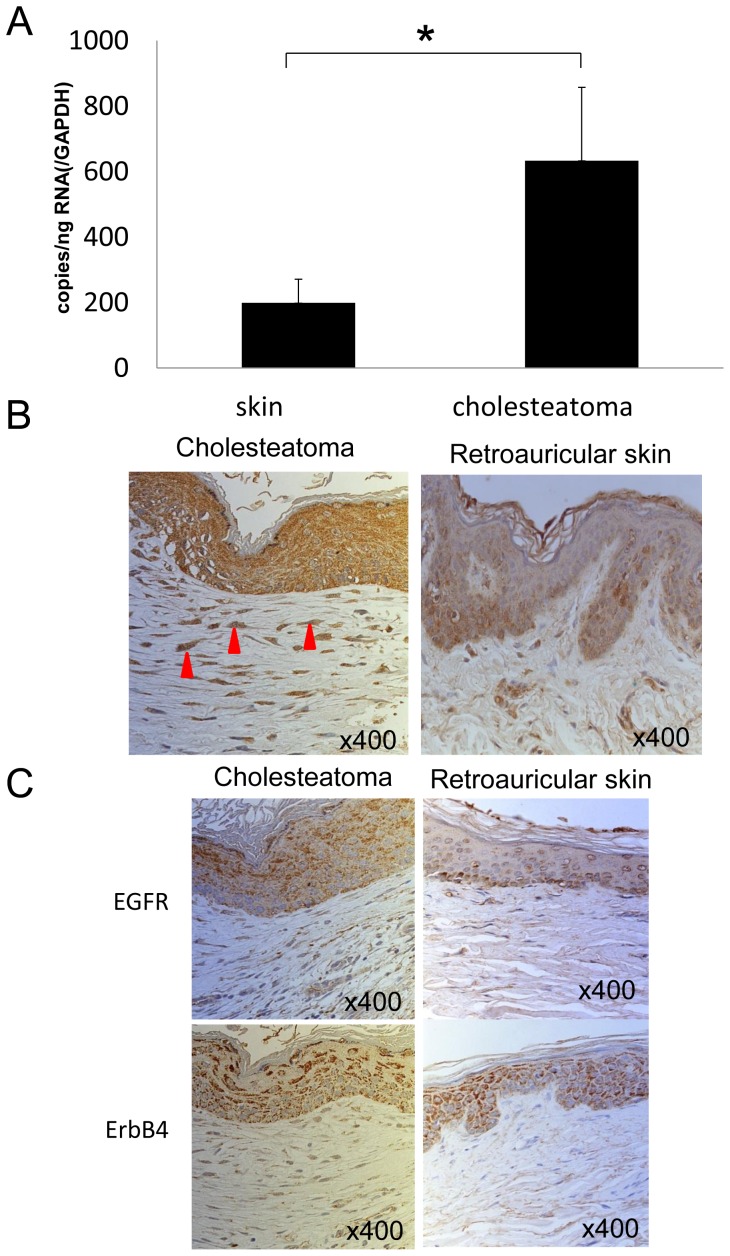
Epiregulin mRNA and protein expression in middle ear cholesteatoma and retroauricular skin. (**A**) *Epiregulin* mRNA expression was significantly higher in cholesteatoma tissue than in retroauricular skin. Data are presented as the mean ± SEM (n = 10). Differences were considered significant at *p*<0.05. (**B**) Epiregulin protein was stained more intensely in epithelial cells and subepithelial fibroblasts (red arrows) from cholesteatoma tissue than in retroauricular skin. (**C**) Staining for EGFR and ErbB4 was strong in the epithelial cells of both cholesteatoma and retroauricular skin.

We assessed the expression of *epiregulin* mRNA by quantitative PCR, and found that it was significantly higher in middle ear cholesteatoma tissue than in normal retroauricular skin (**Figure 1A**).

We also performed immunohistochemistry, which revealed that epiregulin staining was stronger in the epithelial cells and subepithelial fibroblasts of cholesteatoma tissue than in those cells of retroauricular skin. In particular, expression of epiregulin was almost undetectable in the subepithelium of retroauricular skin (**Figure 1B**).

Epiregulin is known to bind with the EGFR and ErbB4. Immunohistochemistry of cholesteatoma tissues and retroauricular skin samples revealed strong expression of both EGFR and ErbB4 by the epithelial cells of both cholesteatoma and retroauricular skin **(** **Figure 1C** **)**.

### Epiregulin Induces Keratinocyte Proliferation via EGFR Signaling

**Figure 2:**
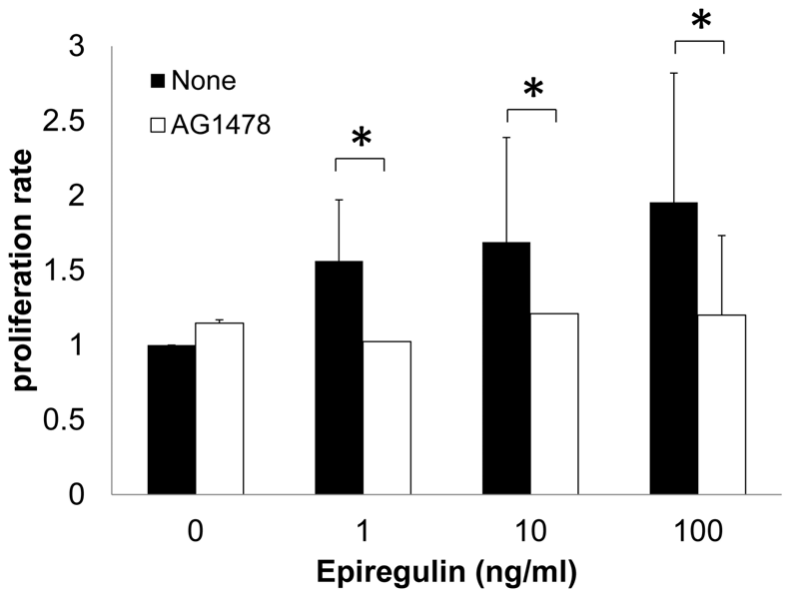
Epiregulin induces keratinocyte proliferation through EGFR signaling. Recombinant human epiregulin (1–100 ng/ml) induced proliferation of PHK16-0b cells in a dose-dependent manner. Induction of proliferation by epiregulin was significantly inhibited by AG-1478, an EGFR tyrosine kinase inhibitor. Data are presented as the mean ± SEM (n = 10). Differences were considered significant at *p*<0.05.

To assess the influence of epiregulin on keratinocyte proliferation, we performed a proliferation assay using PHK16-0b cells (a keratinocyte cell line) and recombinant epiregulin. We found that recombinant human epiregulin (1–100 ng/ml) markedly induced the proliferation of PHK16-0b cells. To examine the role of EGFR in cell proliferation mediated by epiregulin, an EGFR tyrosine kinase inhibitor (AG1478) was added to cells in the proliferation assay. As shown in Figure 2, the induction of cell proliferation by epiregulin was completely inhibited after addition of AG-1478.

### Colony-forming Efficiency of Keratinocytes is Higher after Co-culture with MECF than SF

**Figure 3:**
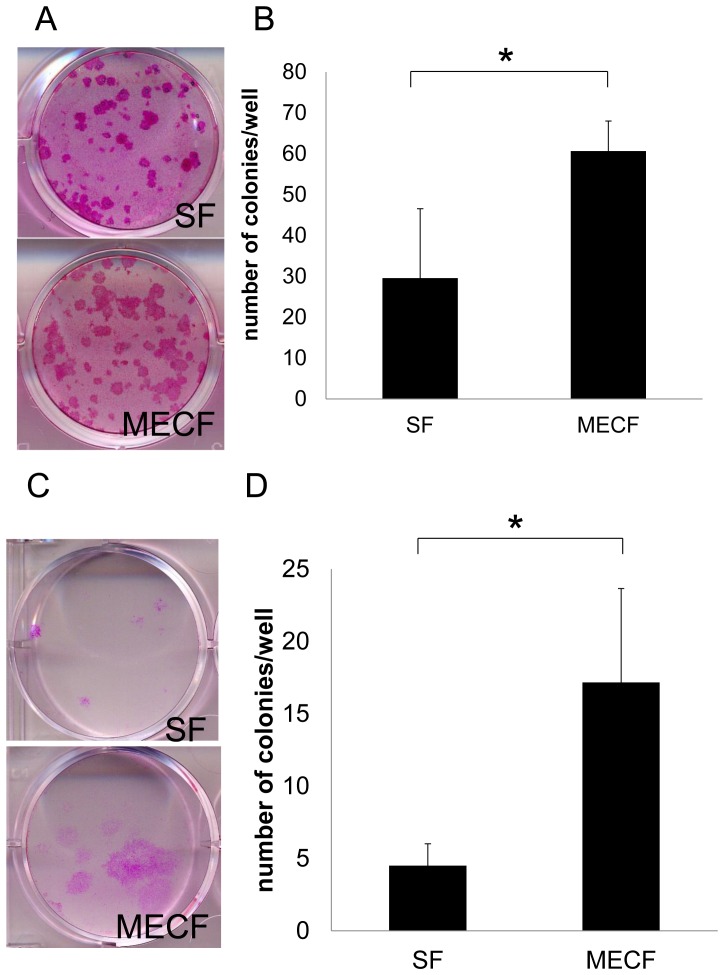
Colony-forming efficiency of keratinocytes co-cultured with MECF or SF. (**A**) Representative picture of colonies formed by PHK16-0b cells cultured with MECF or SF for 21 days, fixed with formalin, and stained with 1% rhodamine B. (***B***) PHK16-0b cells cultured with MECF showed 50% higher colony-forming efficiency than cells cultured with SF. Data are presented as the mean ± SEM (n = 10). (**C**) Representative picture of colonies formed by human epidermal keratinocytes (HEK) cultured with MECF or SF and processed in the same fashion as in (A). Larger colonies were obtained in co-culture with MECF, as in (A). (**D**) HEK cultured with MECF showed higher colony-forming efficiency than HEK cultured with SF. Data are presented as the mean ± SEM (n = 10). Differences were considered significant at *p*<0.05.

To investigate the influence of fibroblasts derived from cholesteatomas on the clonal growth of keratinocytes, the colony-forming assay was performed using PHK16-0b cells co-cultured for 21 days with mitomycin C-treated MECF or SF as feeder cells. PHK16-0b cells cultured with MECF feeders showed a colony-forming efficiency (CFE) than cells cultured with SF, and there was a 50% increase of the CFE **(** **Figures 3A, 3B** **)**. HEK cells also formed colonies during co-culture, although the CFE of HEK cells was relatively low compared with that of PHK16-0b cells **(** **Figures 3A, 3C** **)**. HEK cells cultured with MECF also showed a higher CFE than cells cultured with SF and larger colonies were obtained by co-culture with MECF **(** **Figures 3C, 3D** **)**.

### Colony-forming Efficiency of Keratinocytes is Increased by Epiregulin Overexpression in MECF

**Figure 4:**
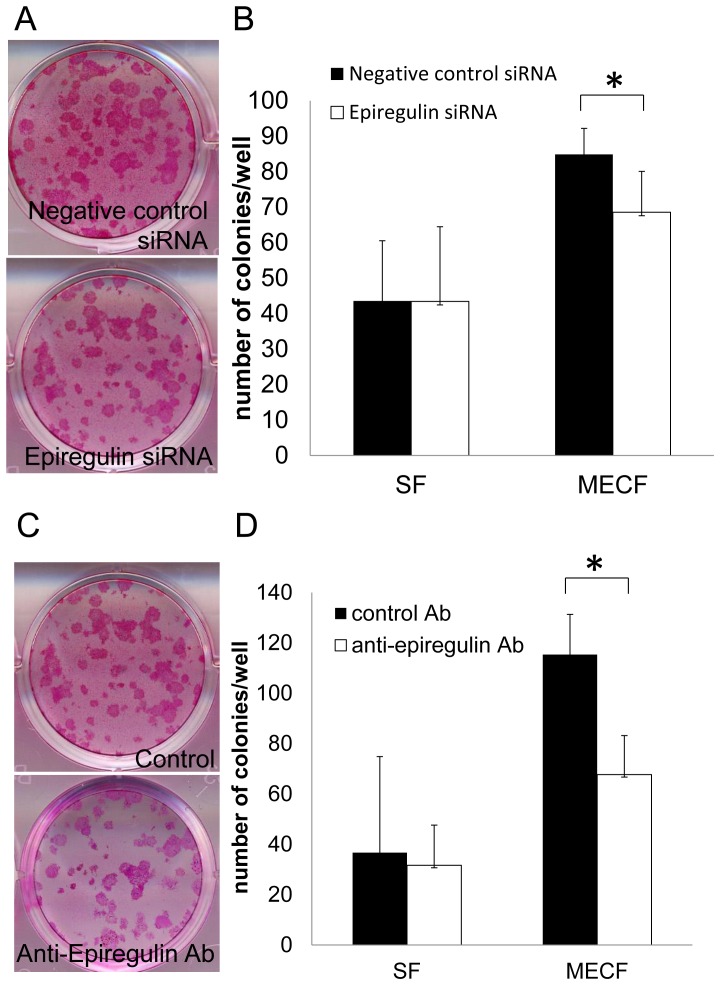
Colony-forming efficiency of keratinocytes is influenced by epiregulin overexpression of in MECF. (**A**) Representative picture of colonies formed by PHK16-0b cells. PHK16-0b cells were cultured with MECF or SF that had been transfected with siRNA, and then were fixed in formalin and stained with 1% rhodamine B. (**B**) PHK16-0b cells co-cultured with MECF transfected with epiregulin siRNA showed significantly lower colony-forming efficiency than PHK16-0b cells co-cultured with MECF transfected with negative siRNA. Data are presented as the mean ± SEM (n = 10). (**C**) Representative picture of colonies formed by PHK16-0b cells cultured with MECF or SF and an epiregulin neutralizing antibody. (**D**) PHK16-0b cells showed significantly lower colony-forming efficiency after co-culture with MECF and the epiregulin neutralizing antibody, but not after co-culture with SF. Data are presented as the mean ± SEM (n = 10). Differences were considered significant at *p*<0.05.

We previously found that the expression of epiregulin mRNA was higher in MECF than in SF. This time, we investigated whether enhanced CFE was mediated through the overproduction of epiregulin by MECF. Initially, we confirmed the effect of silencing epiregulin by transfection of MECF and SF with epiregulin siRNA. When PHK16-0b cells were co-cultured with MECF that had been transfected with epiregulin siRNA, CFE was lower than after co-culture with MECF that had been transfected with negative siRNA, while transfection of SF had no effect (**Figures 4A, 4B**). Next, we investigated the influence of epiregulin protein expressed on the surface of MECF and secreted into the culture medium. PHK16-0b cells were co-cultured with MECF or SF in the presence of an epiregulin neutralizing antibody or a control antibody for the first 7 days and CFE was assessed at 21 days. As shown in **Figures 4C** and 4D, CFE was significantly reduced by treatment with the epiregulin neutralizing antibody when PHK16-0b cells were co-cultured with MECF, but not when these cells were co-cultured with SF. These results indicated that epiregulin has a dominant influence on keratinocyte proliferation through the epithelial-mesenchymal interaction.

## Discussion

Middle ear cholesteatoma is a non-neoplastic accumulation of keratinizing stratified squamous epithelium along with desquamated keratin debris that occurs in the tympanic cavity and mastoid region. Hyperproliferation of keratinocytes has a crucial role in the pathogenesis of cholesteatoma, and it is thought that keratinocyte proliferation is mediated by various autocrine and paracrine growth factors and their receptors [2], [3], [21], [22]. The present study showed that expression of epiregulin was enhanced in the epithelial cells and subepithelial fibroblasts of cholesteatoma tissue compared with normal retroauricular skin. We also found that keratinocyte proliferation and activation occurred in response to co-culture with MECF that overexpressed epiregulin. Furthermore, treatment with an epiregulin neutralizing antibody or epiregulin siRNA significantly reduced the CFE of PHK16-0b cells co-cultured with MECF, but did not affect the CFE when these cells co-cultured with SF.

This study demonstrated the overexpression of epiregulin in epithelial cells and subepithelial fibroblasts of cholesteatoma tissue. Overexpression of epiregulin has already been reported in psoriatic epidermis [16], and a high tissue level of epiregulin is a predictor of a poor prognosis in patients with colorectal carcinoma [23], [24]. These reports suggest that epiregulin is involved in the pathogenesis of various hyperproliferative epithelial diseases.

Epiregulin is an EGF family member that activates EGFR and ErbB4 homodimers and all possible heterodimeric ErbB complexes [15]. A previous study suggested that epiregulin is mitogenic for keratinocytes and stimulates the EGF signaling cascade in these cells via phosphorylation of the EGF receptor [25]. Our results showed that an EGFR tyrosine kinase inhibitor (AG-1478) inhibited the proliferation of PHK16-0b cells and this finding suggested that ErbB signaling also plays an important role in stimulation of keratinocyte proliferation by epiregulin.

In the present study, co-culture with MECF was shown to increase CFE relative to co-culture with SF, but the reason for enhancement of epiregulin expression in MECF is unclear. There have been several reports that fibroblasts from lesions display a different phenotype to those from normal tissues [26], [27]. Epiregulin expression in fibroblasts is up-regulated by IL-1, TNF-α, and EGF [12], [28]. It has also been reported that epithelial–mesenchymal interactions between stromal fibroblasts and cancer cells influence the functional properties of tumor epithelium, with observations on various cancers indicating an important role of stromal fibroblasts in tumor progression and metastasis [29], [30], [31], [32], [33]. Phenotypic changes of fibroblasts may be orchestrated by the combined influence of epigenetic effects including DNA methylation, posttranslational modifications of the histone protein constituents of chromatin, and regulatory noncoding RNAs under the influence of stimuli such as chronic inflammation. Another possibility is that genetic factors regulate the overexpression of epiregulin in MECF. For example, epiregulin is overexpressed by fibroblast-like cells derived from tuberous sclerosis complex skin tumors with allelic deletion of *TSC2* [34]. Thus, further investigation is needed to elucidate the mechanisms underlying phenotypic changes in fibroblasts.

The present study also showed that keratinocyte proliferation was inhibited by an EGFR tyrosine kinase inhibitor. Broad-spectrum anti-ErbB agents, such as lapatinib and canertinib, have been reported to show higher efficacy and wide-ranging antitumor activity [35]. Our findings suggested that anti-ErbB agents may potentially be effective for treating cholesteatoma. However, EGF receptor signaling controls morphogenesis and/or homeostasis in various tissues [36], so local administration would be necessary for a benign disease such as cholesteatoma to avoid systemic side effects. Our experiments showing a decrease of colony formation in the presence of epiregulin neutralizing antibody and epiregulin siRNA indicate that the epithelial-mesenchymal interaction could possibly be controlled in middle ear cholesteatoma, and suggest that epiregulin produced by subepithelial fibroblasts may be a potential target for the treatment of this disease.

In conclusion, our present results suggest that keratinocyte hyperproliferation in middle ear cholesteatoma might be induced through overproduction of epiregulin by subepithelial fibroblasts. Thus, epiregulin is a candidate factor responsible for fibroblast overactivity that influences the epithelium in cholesteatoma.
